# Prognostic Value of Preoperative Peak Expiratory Flow to Predict Postoperative Pulmonary Complications in Surgical Lung Cancer Patients

**DOI:** 10.3389/fonc.2021.782774

**Published:** 2021-11-22

**Authors:** Shuai Chang, Kun Zhou, Yan Wang, Yutian Lai, Guowei Che

**Affiliations:** ^1^ Department of Thoracic Surgery, West China Hospital, Sichuan University, Chengdu, China; ^2^ Department of Thoracic Surgery, The First Affiliated Hospital, Zhejiang University School of Medicine, Hangzhou, China; ^3^ Lung Cancer Center, West China Hospital, Sichuan University, Chengdu, China

**Keywords:** peak expiratory flow (PEF), postoperative pulmonary complications, lung neoplasms, pulmonary surgical procedures, pulmonary rehabilitation

## Abstract

**Objectives:**

Cough impairment may lead to excessive accumulation of pulmonary secretions and increase the risk of postoperative pulmonary complications (PPCs). Peak expiratory flow (PEF) is a sensitive indicator of cough ability. We aimed to investigate the correlation between PEF and PPCs for lung cancer patients undergoing lobectomy or segmental resection for improved risk assessment.

**Methods:**

This retrospective study assessed 560 patients with non-small cell lung cancer admitted for surgery between January 2014 to June 2016. The measurements of PEF were performed before surgery and the clinical outcomes were recorded, including PPCs, postoperative hospital stay, hospitalization costs, and other variables.

**Results:**

Preoperative PEF was significantly lower in PPCs group compared to non-PPCs group (294.2 ± 95.7 vs. 363.0 ± 105.6 L/min, P < 0.001). Multivariable regression analysis showed that high PEF (OR=0.991, 95%CI: 0.988-0.993, P < 0.001) was an independent protective factor for PPCs. According to the receiver operating characteristic (ROC) curve, a PEF value of 250 L/min was selected as the optimal cutoff value in female patients, and 320 L/min in male patients. Patients with PEF under cutoff value of either sex had higher PPCs rate and unfavorable clinical outcomes.

**Conclusions:**

Preoperative PEF was found to be a significant predictor of PPCs for surgical lung cancer patients. It may be beneficial to consider the cutoff value of PEF in perioperative risk assessment.

## Introduction

Lung cancer continues to be the leading cause of cancer death worldwide and poses to be a threat to human health and economic burden (1). Surgical resection with lymph node dissection remains the optimal curative treatment for resectable lung cancer patients (2). However, surgery directly impairs postoperative respiratory function, and the incidence of postoperative pulmonary complications (PPCs) is as high as 19%-59% due to reasons such as reduced lung volume, weakened respiratory muscle strength and reduced cough efficiency (3). As a result, PPCs not only seriously affect the recovery of cardiopulmonary function, but also prolong the length of hospital stay and increase the cost of hospitalization. Consequently, PPC is one of the leading causes of postoperative death and treatment failure (4).

Several clinical studies have defined risk factors for PPC and consensus risk factors including advanced age, poor lung function status, smoking history, chronic obstructive pulmonary disease (COPD), American Society of Anesthesiologists (ASA) score ≥ 3, and long duration of surgery (5–7). Complications such as postoperative pneumonia, atelectasis and pleural effusion resulting in PPC are mostly caused by the reduced efficiency of cough. As a consequence, this may result in excessive accumulation of pulmonary secretions, increasing the risk of airway obstruction and postoperative respiratory infection (8). Peak expiratory flow (PEF) is usually defined as the maximum airflow rate that a person can exhale over a period of 10 milliseconds, which is a sensitive indicator of coughing ability, reflecting airway resistance and respiratory muscle strength (9). Thus, we hypothesized that preoperative PEF would be an effective indicator to predict PPCs that may arise in surgical lung cancer patients.

In the present study, we conducted a prospective cohort study to investigate the correlation between preoperative PEF and clinical outcomes in patients with lung cancer undergoing anatomic lobectomy or segmentectomy.

## Materials and Methods

### Population

A total of 720 surgical lung cancer patients who underwent surgery by the same surgeon was collected in our unit between January 2014 to June 2016. Eligibility for this study were the patients who had a pathological diagnosis of non-small cell lung cancer (NSCLC) and underwent lung cancer anatomic lobectomy or segmentectomy, including open thoracotomy and video-assisted thoracoscopic surgery. Patients were excluded if they: (I) had preoperative signs of pulmonary infection, including purulent sputum or positive signs on sputum microbiology, temperature > 38°C, leukocytosis before surgery; (II) had a history of antibiotic therapy within two weeks before surgery; and (III) had incomplete clinical data. Finally, 560 patients were enrolled in this study, including 104 patients who had PPCs and 456 patients without PPCs. Lung cancer was pathologically staged according to the International Union Against Cancer staging system (8th edition) (10). All patients received resection under general anesthesia and patient-controlled intravenous analgesia for postoperative pain control.

### Peak Expiratory Flow

Prior to surgery, PEF was measured at the bedside with a portable peak flow meter. Patients took standing position and were instructed to inhale deeply, and then exhale as forcefully and quickly as possible. The patients need to complete at least 3 qualified measurements and the highest PEF value was recorded (the difference between the best two results should be within 20 L/min).

### Definition of PPCs

PPCs, as defined earlier (11–13) included (I) pneumonia, chest radiological evidence of new or progressive pulmonary infiltrate and associated with at least one of the following: fever (>38°C), purulent sputum, leukocytosis (>12000/mm^3^) or leukopenia (< 4000/mm^3^), upgraded antibiotic class or extended use time (antibiotics are continued longer than 24 hours postoperatively); (II) atelectasis, part of the lung turns airless and contracts, diagnosed by chest X-ray within 24 hours after surgery; (III) prolonged air leak, postoperative air leak requiring chest tube drainage > 5 days; (IV) pneumothorax, diagnosed by chest X-ray within 24 hours after surgery, air in the pleural space > 30%, and requiring chest tube replacement; (V) pleural effusion, diagnosed by chest X-ray within 24 hours after surgery, pleural effusion requiring thoracocentesis; (VI) bronchospasm, newly detected expiratory wheezing treated with bronchodilators.; (VII) acute respiratory failure, requiring mechanical ventilation > 48h or re-intubation; (VIII) bronchopleural fistula, diagnosed by bronchoscopy; and (IX) pulmonary embolism, diagnosed by pulmo-nary CT angiography.

### Grouping Criterion

First of all, the patients were categorized into those with and without PPC, according to whether they suffered PPCs. To distinguish the variables that correlated with PPCs, between-group comparisons were made. Secondly, the significant variables identified were then included in a multivariable logistic regression analysis to evaluate the independent risk factors of PPCs. Based on the above analysis results, we performed a receiver operating characteristic (ROC) analysis to determine the performance of PEF for predicting PPCs. The ROC curve was conducted in males and females separately due to inherent sex-dependent differences in PEF reference standards. Youden’s Index was selected for the optimal cutoff value of the ROC curve. Lastly, we compared the incidences of PPCs between patients who were demarcated by the PEF cutoff value.

### Outcomes of Interest

The primary outcome of interest was the occurrence of PPCs during hospitalization. The secondary endpoints were the length of postsurgical hospitalization (= discharge date – operation date + 1) and expense incurred during hospitalization.

### Statistical Analysis

Data were analyzed using SPSS v.23.0 and MedCalc v.15.2.2 software. Continuous variables were presented as the mean with standard deviation (mean ± SD), and categorical variables as proportions (n, %). In univariate analyses, continuous variables were compared by Student’s t-test and categorical variables were analyzed using Pearson’s chi-squared or Fisher’s exact test. Independent risk factors of PPCs were identified by using PEF and other variables with P < 0.1 as inputs for a multivariable binary logistic regression model. The discriminative power of PEF on predictions for PPCs was evaluated by ROC analysis, followed by calculation of the area under curve (AUC). All results were considered statistically significant at P < 0.05.

## Results

### Study Population and Characteristics

A total of 560 patients who met eligibility criteria were invited to participate in this study, with 104 patients occurred PPCs in 30 days after the operation, who were divided into PPCs group. The baseline characteristics between the two groups are listed in **Table 1**.

**Table 1:** Baseline and clinical characteristics between the PPCs groups and non-PPCs group.

Variables	PPCs group (*n* = 104)	non-PPCs group (*n* = 456)	*P*-value
Age (years), mean ± SD	64.6 ± 8.9	61.0 ± 8.6	<0.001
BMI (kg/m^2^), mean ± SD	23.3 ± 3.1	23.4 ± 2.9	0.667
Gender, *n* (%)			0.063
Male	67 (64.4%)	248 (54.4%)	
Female	37 (35.6%)	208 (45.6%)	
Pulmonary function, mean ± SD			
PEF (L/min)	294.2 ± 95.7	363.0 ± 105.6	<0.001
FEV1 (L)	2.0 ± 0.6	2.4 ± 0.7	<0.001
FEV1%	86.5 ± 23.6	99.0 ± 20.8	<0.001
Smoking status, *n* (%)			<0.001
Current or former smokers	71 (68.3%)	210 (46.1%)	
Non-smokers	33 (31.7%)	246 (53.9%)	
Comorbidities, *n* (%)			
Diabetes	15 (14.4%)	49 (10.7%)	0.287
Hypertension	30 (28.8%)	120 (26.3%)	0.599
COPD	46 (46.9%)	105 (25.1%)	<0.001
Surgical approach, *n* (%)			<0.001
Open	41 (39.4%)	94 (20.6%)	
VATS	63 (60.6%)	362 (79.4%)	
Resection type, *n* (%)			0.002
Lobectomy	78 (75.0%)	267 (58.6%)	
Sublobar resection	26 (25.0%)	189 (41.4%)	
Operation time (min), mean ± SD	142.7 ± 49.6	112.4 ± 47.2	<0.001
Pathological type, *n* (%)			0.123
Adenocarcinoma	56 (53.9%)	286 (62.7%)	
Squamous carcinoma	33 (31.7%)	102 (22.4%)	
Other NSCLC	15 (14.4%)	68 (14.9%)	
Pathological stage, *n* (%)			0.712
Stage I	59 (56.7%)	276 (60.5%)	
Stage II	26 (25.0%)	98 (21.5%)	
Stage III+IV	19 (18.3%)	82 (18.0%)	
Postoperative stay, mean ± SD	9.8 ± 4.1	5.6 ± 2.2	<0.001
Hospitalization expenses ($), mean ± SD	9,359 ± 2,134	7,305 ± 1,884	<0.001

BMI, body mass index; PEF, peak expiratory flow; FEV1, forced expiratory volume in 1 second; COPD, chronic obstructive pulmonary disease; VATS, video-assisted thoracoscopic surgery; NSCLC, non–small cell lung cancer.

The mean age of patients in the PPCs group was significantly higher than those in the non-PPCs group (64.6 ± 8.9 vs. 61.0 ± 8.6 yr, P < 0.001). Significantly lower PEF (294.2 ± 95.7 vs. 363.0 ± 105.6 L/min, P < 0.001), forced expiratory volume in one second (FEV1; 2.0 ± 0.6 vs. 2.4 ± 0.7 L, P < 0.001), and FEV1% (86.5 ± 23.6 vs. 99.0 ± 20.8, P < 0.001) were found in PPCs group. The proportion of smokers (68.3% vs. 46.2%, P < 0.001), chronic obstructive pulmonary disease (COPD; 46.9% vs. 25.1%, P < 0.001) and open thoracotomy (39.4% vs. 20.6%, P < 0.001) were higher in PPCs group compared to that in non-PPCs group. Additionally, the patients in PPCs group showed significantly longer postoperative stay (9.8 ± 4.1 vs. 5.6 ± 2.2 days, P < 0.001) and greater hospitalization expenses ($9,359 ± $2,135 vs. $7,305 ± $1,884; P < 0.001).

### Multivariable Analysis of Risk Factors for PPCs

The variables with P < 0.1 in univariate analysis including age, gender, PEF, FEV1, FEV1%, smoking history, COPD, open thoracotomy and duration of surgery, were included in a multivariable regression model. The multivariable analysis showed that high PEF (OR = 0.991, 95%CI: 0.988-0.993, P < 0.001) was a protective factor. Smoking (OR = 4.136, 95% CI: 2.373-7.301, P < 0.001), video-assisted thoracoscopic surgery (OR = 0.560, 95% CI: 0.329-0.956, P = 0.034) and duration of surgery > 3h (OR = 3.903, 95% CI: 1.998-7.625, P < 0.001) could independently predict the occurrence of PPCs. Univariable and multivariable analyses are shown in **Table 2**.

**Table 2:** Univariate and multivariable analysis for risk factors of PPCs.

Variables	Category	Univariate analysis			Multivariable analysis		
		OR	95% CI	*P*-value	OR	95% CI	*P*-value
Age	<70, ≥70	2.089	1.291-3.380	0.003	1.746	0.951-3.205	0.072
Gender	F, M	1.519	0.976-2.362	0.064	0.469	0.162-1.359	0.163
PEF	Per unit increase	0.993	0.990-0.995	<0.001	0.991	0.987-0.995	<0.001
PEF(male)	≥320,<320	4.667	2.643-8.241	<0.001	–	–	–
PEF(female)	≥250,<250	4.929	2.364-10.276	<0.001	–	–	–
FEV1	Per unit increase	0.438	0.306-0.627	<0.001	1.392	0.718-2.698	0.328
FEV1%	≥70, <70	4.170	2.429-7.159	<0.001	2.145	0.957-4.809	0.064
Lobectomy	Yes	2.124	1.132-3.437	0.002	1.604	0.918-2.803	0.097
Smoking status	Yes	2.510	1.597-3.945	<0.001	5.457	1.980-15.038	0.001
Diabetes	Yes	1.400	0.751-2.608	0.289	–	–	–
Hypertension	Yes	1.135	0.708-1.821	0.599	–	–	–
COPD	Yes	2.637	1.674-4.153	<0.001	1.198	0.642-2.235	0.570
VATS procedure	Yes	0.399	0.253-0.628	<0.001	0.542	0.316-0.929	0.026
Operation time	<3 h, ≥3 h	2.805	1.582-4.974	<0.001	3.529	1.840-6.769	<0.001

PEF, peak expiratory flow; FEV1, forced expiratory volume in 1 second; COPD, chronic obstructive pulmonary disease; VATS, video-assisted thoracoscopic surgery.

### ROC Analysis of the Prediction of PEF for PPCs

**Figure 1:**
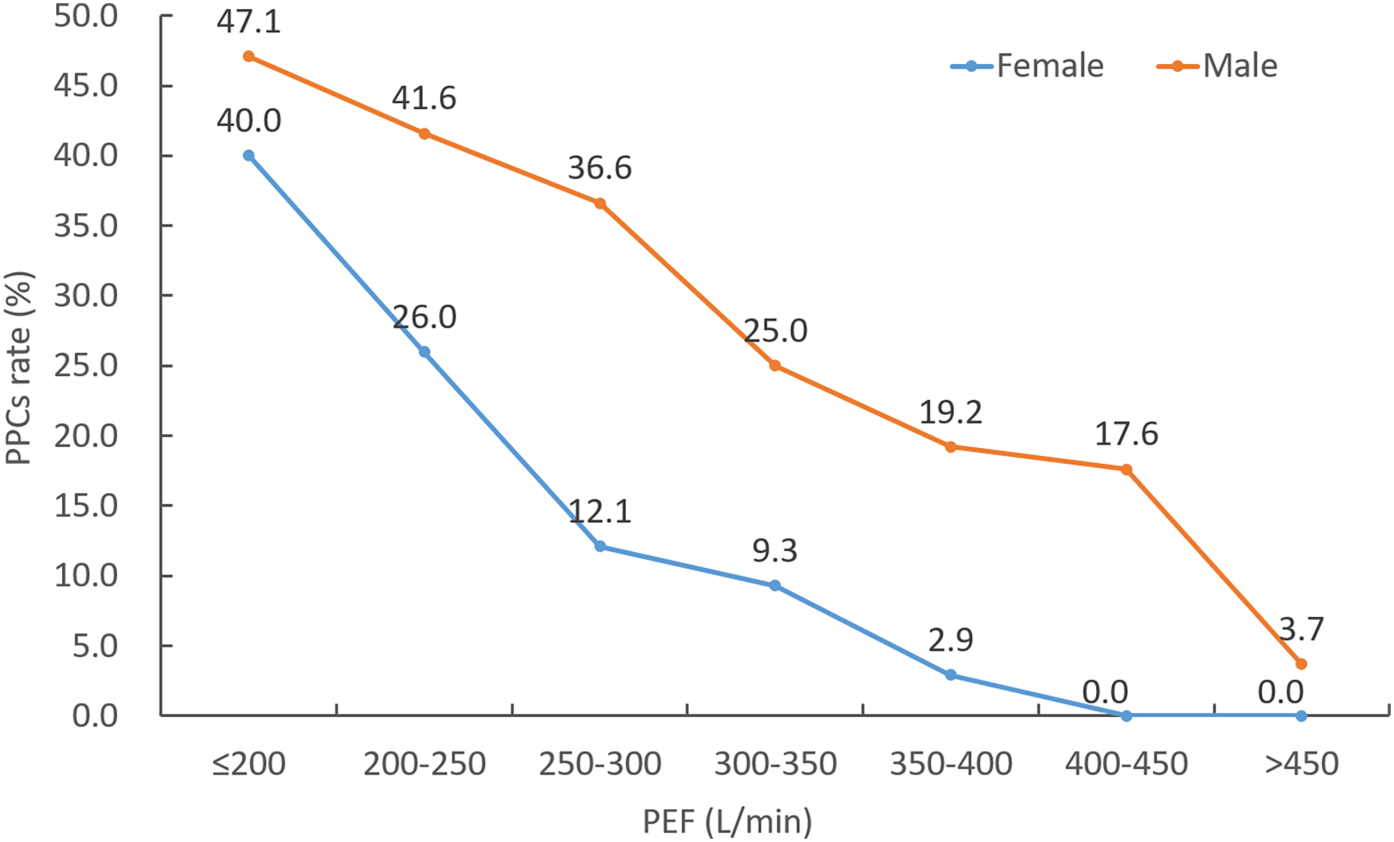
The incidence of PPCs in different ranges of PEF.

**Figure 2:**
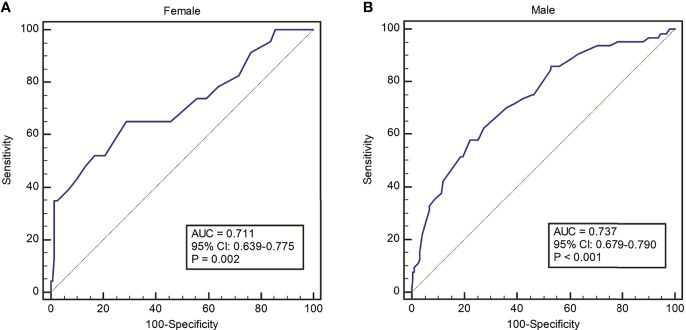
ROC analysis on discriminative power of PEF for predicting risk of PPCs in **(A)** female and **(B)** male groups.

The incidence of PPCs with varying distributions of PEF is shown in **Figure 1**. A trend towards decreasing rate of PPCs with increasing PEF value was observed. The ROC analysis of PEF showed an AUC of 0.711 (95% CI: 0.639-0.775, P = 0.002) in female patients and an AUC of 0.737 (95% CI: 0.679-0.790, P < 0.001) in male patients for predicting PPCs (**Figure 2**). According to the ROC curve, a PEF value of 250 L/min was selected as the optimal cutoff value for predicting PPCs in the female group (Youden index: 0.364, sensitivity: 65.2%, specificity: 71.3%), whilst a PEF value of 320 L/min was the cutoff value in the male group (Youden index: 0.356, sensitivity: 57.8%, specificity: 77.8%) (**Figure 2**).

### Comparisons Between Patients Demarcated by PEF Cutoff Value

#### Female Group

Among 245 female patients, PEF ≤ 250 L/min was confirmed in 75 patients (ratio = 30.6%). Patients in PEF ≤ 250 L/min group was older (65.4 ± 7.8 vs. 59.3 ± 8.9 yr, P < 0.001) and had worse physical conditions. Their pulmonary function indicators of FEV1 and FEV1% were significantly lower than those of patients in PEF > 250 L/min group. Moreover, a higher percentage of diabetes, hypertension, and COPD were found in PEF ≤ 250 L/min group. With regard to clinical outcomes, patients with PEF ≤ 250 L/min had longer postoperative stay (7.0 ± 3.1 vs. 5.5 ± 2.7 days, P = 0.001) and higher hospitalization expenses ($7,704 ± $2,136vs. $7,117 ± $2,075, P = 0.045), drug costs ($1,228 ± 568 vs. $977 ± 499, P = 0.001), and PPCs rate (30.7% vs. 8.2%, P < 0.001) (**Table 3**).

**Table 3:** Clinical characteristics and outcomes between female patients divided by cutoff value of PEF.

Variables	PEF ≤ 250 L/min (*n* = 75)	PEF > 250 L/min (*n* = 170)	*P*-value
Age (years), mean ± SD	65.4 ± 7.8	59.3 ± 8.9	<0.001
BMI (kg/m^2^), mean ± SD	23.6 ± 3.5	23.1 ± 2.9	0.297
Pulmonary function, mean ± SD			
PEF (L/min)	214.1 ± 37.8	337.7 ± 50.8	<0.001
FEV1 (L)	1.6 ± 0.4	2.1 ± 0.4	<0.001
FEV1%	91.6 ± 24.1	108.4 ± 18.3	<0.001
Smoking history, *n* (%)	6 (8.0%)	6 (3.5%)	0.138
Comorbidities, *n* (%)			
Diabetes	13 (17.3%)	13 (7.6%)	0.023
Hypertension	29 (38.7%)	39 (22.9%)	0.011
COPD	28 (40.6%)	12 (7.1%)	<0.001
Surgical approach, *n* (%)			0.164
Open	18 (24.0%)	28 (16.5%)	
VATS	57 (76.0%)	142 (83.5%)	
Operation time (min), mean ± SD	123.4 ± 46.8	113.3 ± 47.2	0.123
Postoperative stay, mean ± SD	7.0 ± 3.1	5.5 ± 2.7	0.001
Hospitalization expenses ($), mean ± SD	7,704 ± 2136	7,117 ± 2,075	0.045
Drug cost ($), mean ± SD	1,228 ± 568	977 ± 499	0.001
PPCs rate, *n* (%)	23 (30.7%)	14 (8.2%)	<0.001
Pneumonia	15 (20.0%)	9 (5.4%)	<0.001
Atelectasis	6 (8.0%)	5 (2.9%)	0.153
Air leak	3 (4.0%)	4 (2.4%)	0.766
Pneumothorax	1 (1.3%)	3 (1.8%)	0.763
Pleural effusion	0 (0.0%)	2 (1.2%)	1.000
Mechanical ventilation >48 h	1 (1.3%)	0 (0.0%)	1.000
Bronchopleural fistula	0 (0.0%)	0 (0.0%)	1.000
Pulmonary embolism	0 (0.0%)	1 (0.6%)	1.000

BMI, body mass index; PEF, peak expiratory flow; FEV1, forced expiratory volume in 1 second; COPD, chronic obstructive pulmonary disease; VATS, video-assisted thoracoscopic surgery.

#### Male Group

Of the 315 male patients, PEF ≤ 320 L/min was confirmed in 96 patients. Patients in PEF ≤ 320 L/min group were older (mean age, 64.7 ± 8.3 vs. 60.8 ± 8.8 yr, P < 0.001), comprised a higher proportion of cases of COPD (66.7% vs. 21.5%, P < 0.001), and had poorer lung function, compared to the PEF > 320 L/min group; however, there were no differences in diabetes and hypertension. Further, with regard to clinical outcomes, patients with PEF ≤ 320L/min had significantly prolonged postoperative stay (8.8 ± 3.9 vs. 6.9 ± 3.1 days, P = 0.001), higher hospitalization costs ($8,294 ± $2,174 vs. $7,804 ± $1,926, c< 0.001) and drug costs ($1,726±$699 vs. $1,299±$582, P <0.001), higher rate of PPCs (40.6% vs. 12.8%, P < 0.001), which were mainly due to significant differences of pneumonia (31.3% vs. 9.1%, P < 0.001) and air leak (12.5% vs. 5.5%, P = 0.031) (**Table 4**).

**Table 4:** Clinical characteristics and outcomes between male patients divided by cutoff value of PEF.

Variables	PEF ≤ 320 L/min (*n* = 96)	PEF > 320 L/min (*n* = 219)	*P*-value
Age (years), mean ± SD	64.7 ± 8.3	60.8 ± 8.8	<0.001
BMI (kg/m^2^), mean ± SD	23.1 ± 2.8	23.6 ± 2.8	0.142
Pulmonary function, mean ± SD			
PEF (L/min)	261.7 ± 47.9	445.3 ± 83.2	<0.001
FEV1 (L)	2.0 ± 0.5	2.8 ± 0.6	<0.001
FEV1%	74.2 ± 18.3	99.3 ± 16.8	<0.001
Smoking history, *n* (%)	87 (90.6%)	182 (83.1%)	0.082
Comorbidities, *n* (%)			
Diabetes	12 (12.5%)	26 (11.9%)	0.875
Hypertension	24 (25.0%)	58 (26.5%)	0.782
COPD	64 (66.7%)	47 (21.5%)	<0.001
Surgical approach, *n* (%)			0.010
Open	25 (26.0%)	64 (29.2%)	0.564
VATS	71 (74.0%)	155 (71.8%)	
Operation time (min), mean ± SD	124.7 ± 54.8	117.2 ± 51.4	0.256
Postoperative stay, mean ± SD	8.8 ± 3.9	6.9 ± 3.1	<0.001
Hospitalization expenses ($), mean ± SD	8,294 ± 2,174	7,804 ± 1,926	<0.001
Drug cost($)	1,726 ± 699	1,299 ± 582	<0.001
PPCs rate, *n* (%)	39 (40.6%)	28 (12.8%)	<0.001
Pneumonia	30 (31.3%)	20 (9.1%)	<0.001
Atelectasis	8 (8.3%)	9 (4.1%)	0.126
Air leak	12 (12.5%)	12 (5.5%)	0.031
Pneumothorax	4 (4.2%)	5 (2.3%)	0.578
Pleural effusion	4 (4.2%)	3 (1.4%)	0.256
Mechanical ventilation >48 h	1 (1.0%)	1 (0.5%)	1.000
Bronchopleural fistula	0 (0.0%)	1 (0.5%)	1.000
Pulmonary embolism	2 (2.1%)	0 (0.0%)	1.000

BMI, body mass index; PEF, peak expiratory flow; FEV1, forced expiratory volume in 1 second; COPD, chronic obstructive pulmonary disease; VATS, video-assisted thoracoscopic surgery.

## Discussion

Pulmonary complications are the principal factors affecting postoperative rehabilitation of lung cancer patients. In addition to optimizing the perioperative process, key objectives of postoperative rapid lung rehabilitation are the prevention and reduction of postoperative pulmonary complications. Therefore, the first step to promote rapid postoperative recovery of lung cancer patients is to identify and evaluate the risk factors of PPCs that enable reasonable intervention. These risk factors vary due to inconsistent definitions and standards of PPCs in various studies, differences in inclusion and exclusion criteria, ethnic and cultural differences, and treatment of complications. Advanced age, smoking history, COPD and reduced lung function have been consistently identified as risk factors in several studies (14–16). Prior to surgery, examination of pulmonary function aids in assessing the risk of pulmonary surgery (17, 18). The perioperative utility of PEF remains debatable since it is mostly used for the diagnosis and follow-up of asthma (19), with relatively few applications in pulmonary surgery. A significant finding of our study was that low PEF values independently predicted the occurrence of PPCs for lung cancer patients undergoing resection. Both female and male patients with PEF under cutoff value reported adverse clinical outcomes, including higher PPCs rate, prolonged length of stay, and increased hospital costs.

The respiratory muscles force and the severity of airway obstruction are closely related to PEF, which can be measured easily, using a portable mechanical or electronic flow meter that is easy to operate and cost-effective. It can be used at home or by the patient’s bedside and only requires the examinee to exhale quickly and forcefully. The process of detecting PEF is similar to coughing, wherein rapid and powerful contraction of the diaphragm and abdominal muscle increase intra-abdominal pressure. Since abdominal contents are virtually incompressible, the volume of the abdominal cavity changes little. The diaphragm then lifts and compresses the chest cavity, and the intercostal muscle retracts, rapidly reducing the volume of the chest cavity, extruding lungs to form high-pressure gas resulting in rapid exhalation (20). Cough is an effective self-protection method for clearing respiratory secretions, and it is also an essential auxiliary means to eliminate pleural effusion and pneumatosis after lung surgery (8, 21). In recent years, advances in and popularity of thoracoscopic surgery has reduced surgical chest wall trauma, but patients with weak respiratory muscles strength who have had lung resection exhibit clinical issues such as poor coughing ability and efficiency, resulting in sputum retention that leads to pulmonary complications. Additionally, lung cancer patients with COPD reach up to 40%-70%. These patients have hypersecretion of mucous glands in the respiratory tract. Meanwhile, the anesthetic drugs and tracheal intubation stimulation could increase airway secretions. The combined effect of coughing impairment and hypersecretion of airway increases the risk of pulmonary infection (22).

Prior to our study, the correlation between PEF and PPCs was controversial. The British Thoracic Society guideline for the physiotherapy management of the spontaneously breathing patient suggests that when the PEF of patients with neuromuscular disease equal to or less than 270 ml/L, the strategies for assisted airway clearance should be used (23). Kulnik et al. found that the strong cough (with high peak cough flow) could protect from aspiration-related pneumonia in patients with stroke and swallowing problems (24). However, Colucci and colleagues drew a negative conclusion that there was no association between the PEF and PPCs in patients who underwent open upper abdominal surgery (8). Our results showed that the PEF of surgical lung cancer patients with PPCs was significantly lower compared to those without PPCs, and low PEF was an independent risk factor for PPCs in the multivariable analysis. We recommend separate gender-based analysis of PEF since it is significantly affected by skeletal muscle strength. The ROC curve calculated that the optimal cutoff value for predicting PPC by PEF was 320 L/min for male patients and 250 L/min for female patients. If the PEF was lower than the cutoff values, PPCs were significantly likely to occur after surgery, both in male and female patients, compared to patients with PEF higher than the cutoff value. Meanwhile, postoperative hospitalization days and hospitalization costs were significantly higher in the low PEF group, compared to high PEF group. Our study provides a new perspective for screening high-risk patients before lung cancer surgery based on these results. Ishida et al. reported that the thickness of the external oblique muscle might be associated with PEF (20), which may help in improving PEF, through such exercise training modalities as oblique crunch and side bridge. These related exercises could be considered as a part of pulmonary rehabilitation program to enhance the respiratory strength and cough efficiency.

### Limitations

This study is not without certain limitations. First, there is no standard definition of PPCs, which may have led to biased results. Second, since all lung cancer resections were performed at a single center, the general applicability of our findings is limited. Third, the variability and accuracy of PEF is closely related to skill proficiency and different spirometers, which requires the technician to strictly check data collection to ensure the authenticity and stability of data. Fourth, the PEF cutoff values in this study were measured in Asians only, thus the generalizability of the results to other races will need investigation. Our results need to be confirmed in prospective studies at multiple centers and with large sample sizes.

## Conclusions

Our prospective cohort study demonstrated that low PEF serves as an independent risk predictor of PPCs for lung cancer patients undergoing lobectomy and segmentectomy. Therefore, considering PEF cutoff value in the perioperative risk assessment for lung cancer patients may be of benefit.

## Data Availability Statement

The raw data supporting the conclusions of this article will be made available by the authors, without undue reservation.

## Ethics Statement

The studies involving human participants were reviewed and approved by Regional Ethics Committee of Sichuan University West China Hospital (No. 2016-121). The registration number was ChiCTR-COC-17010720, which was obtained from the Chinese Clinical Trial Registry. The study adhered to the tenets of the Declaration of Helsinki. The patients/participants provided their written informed consent to participate in this study.

## Author Contributions

GC, YW, and YL contributed to the conception, design of the study. SC and KZ contributed equally as first authors. All authors contributed to the intellectual conception revision of important intellectual content, and approval of the final version of this manuscript.

## Funding

This study was supported by Chengdu Science and Technology Support Program (2019-YF05-00373-SN).

## Conflict of Interest

The authors declare that the research was conducted in the absence of any commercial or financial relationships that could be construed as a potential conflict of interest.

## Publisher’s Note

All claims expressed in this article are solely those of the authors and do not necessarily represent those of their affiliated organizations, or those of the publisher, the editors and the reviewers. Any product that may be evaluated in this article, or claim that may be made by its manufacturer, is not guaranteed or endorsed by the publisher.
